# The prognostic value of the direct bilirubin to albumin ratio in critically ill patients with cirrhosis: Insights from MIMIC-IV database

**DOI:** 10.1371/journal.pone.0334591

**Published:** 2025-10-13

**Authors:** XingYi Yang, GuangDong Wang, Zhang Min, LiHong Lv, Ji Yang

**Affiliations:** 1 Department of Gastroenterology Disease, XianJu People’s Hospital, Zhejiang Southeast Campus of Zhejiang Provincial People’s Hospital, Affiliated Xianju’s Hospital, Hangzhou Medical College, Xianju, Zhejiang, China; 2 Department of Respiratory and Critical Care Medicine, First Affiliated Hospital of Xi’an Jiao tong University, Xi an, Shanxi, China; Dalin Tzu Chi Hospital, Buddhist Tzu Chi Medical Foundation, TAIWAN

## Abstract

**Background:**

Patients with severe cirrhosis are at a higher risk of mortality. This study aimed to investigate the association between the direct bilirubin-to-albumin ratio (DBAR) and 28-day mortality in critically ill cirrhotic patients using data from the publicly available MIMIC-IV database.

**Methods:**

This study explores DBAR’s relationship with 28-day mortality in severe cirrhosis patients. We first conducted univariate and multivariate analyses to identify independent risk factors. Then, we used Kaplan-Meier (KM) survival analysis to assess DBAR’s link with survival time and created KM curves. DBAR’s predictive accuracy was evaluated using Receiver Operating Characteristic (ROC) analysis, and the relationship was examined using restricted cubic spline modeling and subgroup analyses.

**Result:**

This study enrolled 509 cirrhotic patients with in-hospital and ICU mortality rates of 22.3% and 14.3%, respectively. Univariate and multivariate analyses revealed a significant association between DBAR and 28-day mortality risk, with a hazard ratio of 1.16 (95% CI: 1.10–1.24, p < 0.001), confirming DBAR as an independent risk factor for short-term prognosis. DBAR demonstrated good predictive accuracy for 28-day mortality (AUC = 0.702, 95% CI: 0.650–0.753). Patients were divided into low-risk (DBAR < 4) and high-risk (DBAR ≥ 4) groups, with the high-risk group showing a hazard ratio of 3.05 (95% CI 1.87–4.97, p < 0.001) after multivariate adjustment. Restricted cubic spline (RCS) analysis identified a nonlinear relationship between DBAR and 28-day prognosis (p-Nonlinear = 0.022, p < 0.001). Subgroup analysis showed no interaction between DBAR and most subgroups.

**Conclusion:**

The DBAR scoring system offers an efficient and user-friendly approach for assessing prognosis in critically ill cirrhotic patients.

## 1 Introduction

Cirrhosis, the terminal pathological manifestation of various chronic liver diseases, is characterized by persistent hepatocellular necrosis, excessive fibrous tissue deposition, and nodular parenchymal regeneration within the hepatic architecture [1,2]. These progressive structural alterations fundamentally disrupt the liver’s lobular organization and vascular configuration, precipitating severe clinical consequences, particularly portal hypertension and hepatic decompensation, which significantly elevate both morbidity and mortality risks [3,4]. The admission of cirrhotic patients to intensive care units (ICUs) represents a critical clinical turning point, universally associated with poor prognosis [5,6]. This vulnerable population demonstrates increased predisposition to life-threatening complications, notably hepatic encephalopathy and septic events, which substantially complicate therapeutic interventions and markedly escalate mortality rates [7,8].

In the prognostic assessment of cirrhosis, both the Child-Pugh classification system [9] and the Model for End-Stage Liver Disease (MELD) score [10], despite their widespread clinical application, demonstrate significant limitations. The Child-Pugh system, in particular, is constrained by its dependence on subjective clinical parameters, especially in the assessment of hepatic encephalopathy, which reduces its reliability and consistency. This underscores an urgent need for the development of more precise, objective, and minimally invasive prognostic markers to improve predictive accuracy and clinical decision-making in cirrhotic patients.

Total bilirubin and albumin serve as critical biochemical markers in the prognostic evaluation of cirrhosis. Elevated bilirubin levels correlate with cholestatic progression and hepatocyte injury [11], whereas decreased albumin levels signify impaired hepatic synthetic function [12]. The bilirubin-albumin score has established prognostic utility in hepatic encephalopathy and hepatocellular carcinoma [13–15], with the indirect bilirubin/albumin ratio particularly demonstrating predictive accuracy for hepatic encephalopathy development [16]. Emerging evidence suggests that direct bilirubin exhibits superior prognostic value compared to total bilirubin levels in cirrhotic patients [17], highlighting the potential significance of the DBAR as a novel prognostic indicator. The link between DBAR and mortality in critically ill individuals with cirrhosis admitted to the ICU has not yet been thoroughly examined. To fill this research void, we performed a retrospective study utilizing clinical records from the MIMIC-IV v2.2 database [18], focusing on cirrhotic individuals. This study seeks to assess whether DBAR levels are connected to short-term mortality from all causes in this patient population.

## 2 Materials and methods

### 2.1 Database introduction

We utilized the MIMIC-IV database (version 2.2), a publicly accessible critical care dataset curated by the MIT Laboratory for Computational Physiology. The database contains detailed information on more than 70,000 ICU admissions at Beth Israel Deaconess Medical Center from 2012 to 2019. Ethical approval was granted by the Institutional Review Board of Beth Israel Deaconess Medical Center (protocol 2001P-001699/14). As all data were fully de-identified, further institutional approval and individual informed consent were waived.

### 2.2 Inclusion and exclusion criteria

**Fig 1:**
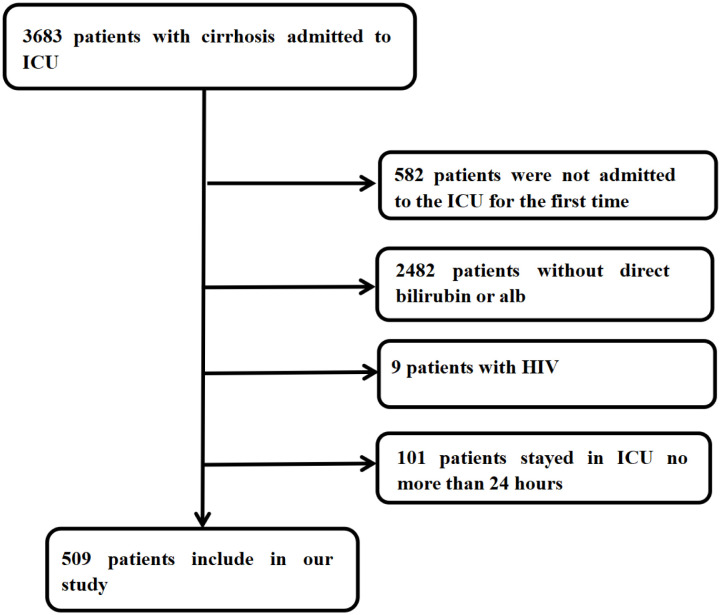
Flow diagram of study participants.

In this retrospective cohort study, patients diagnosed with cirrhosis were included. The diagnosis of cirrhosis was determined by International Classification of Diseases codes. The study included 3683 patients with cirrhosis. Patients were excluded if they were under 18 years old, with HIV, spent less than 24 hours in the ICU, lacked direct bilirubin and albumin data within 24 hours of admission, or had previous ICU admissions. A total of 509 patients were ultimately included in the study. (Further details are delineated in Fig 1)

### 2.3 Data collection and monitoring

Data from the database were extracted, encompassing demographic information, initial clinical vital signs, laboratory results, comorbidities, and treatment outcomes, with a specific focus on the first 24 hours post-ICU admission. Key vital signs monitored included blood pressure, heart rate, respiratory rate, and body temperature. Comprehensive laboratory parameters were recorded, including blood glucose, white blood cell count (WBC), international normalized ratio (INR), platelet count, sodium, potassium, blood urea nitrogen (BUN), aminotransferase (ALT), and aspartate aminotransferase (AST), creatinine, direct bilirubin, albumin, and complications. Significant attention was devoted to the collection of data pertaining to complications associated with cirrhosis, including acute kidney injury (AKI), ascites, variceal hemorrhage, hepatorenal syndrome (HRS), and spontaneous bacterial peritonitis (SBP), alongside prevalent therapeutic interventions such as continuous renal replacement therapy (CRRT) and vasopressor administration. The DBAR was selected as the primary study variable, calculated as the average of values recorded within the initial 24 hours of ICU admission for all patients. The study cohort comprised individuals who met established diagnostic criteria for cirrhosis and were followed for a minimum of 28 days to evaluate short-term outcomes. Table 1 outlines the complete list of extracted variables in detail. The primary endpoint was defined as all-cause mortality at 28 days post-admission.

**Table 1:** Patient demographics and baseline characteristics.

Variables	All patients (n = 509)	Survivors (n = 375)	Non-survivors (n = 134)	p-value
**Demographic**				
Age (years)	59 (52, 66)	58 (51, 65)	60 (52, 69)	0.072
Gender, n (%)				0.102
Male	355 (69.7%)	269 (71.7%)	86 (64.2%)	
Female	154 (30.3%)	106 (28.3%)	48 (35.8%)	
Race, n (%)				0.067
White	334 (65.6%)	254 (67.7%)	80 (59.7%)	
Other	175 (34.4%)	121 (32.3%)	54 (40.3%)	
**Vital Signs**				
Heart rate (beats/minute)	91 (79, 103)	90 (79, 102)	94 (81, 109)	0.019
Systolic blood pressure (mmHg)	117 (102, 133)	120 (106, 136)	111 (94, 123)	<0.001
Diastolic Blood Pressure (mmHg)	62 (53, 71)	62 (55, 71)	61 (51, 69)	0.124
Respiratory rate (beats/min)	18.0 (15.0, 22.0)	17.0 (15.0, 20.0)	19.5 (16.0, 25.0)	<0.001
Temperature (°C)	36.83(36.50, 37.20)	36.89 (36.56, 37.28)	36.61(36.22, 36.94)	<0.001
**Laboratory Indicators**				
Bicarbonate (m Eq/l)	21.0 (18.0, 25.0)	22.0 (19.0, 25.0)	19.5 (16.0, 23.0)	<0.001
Anion Gap (m Eq/l)	16.0 (13.0, 19.0)	16.0 (13.0, 19.0)	17.0 (15.0, 21.0)	<0.001
Ca (mg/dl)	8.30 (7.70, 9.00)	8.30 (7.80, 9.10)	8.20 (7.50, 8.90)	0.019
BUN (mg/dl)	23 (15, 44)	20 (14, 34)	39 (22, 65)	<0.001
Potassium (m Eq/l)	4.10 (3.70, 4.70)	4.10 (3.70, 4.70)	4.30 (3.70, 5.10)	0.031
Sodium (m Eq/l)	138 (133, 141)	138 (135, 141)	134 (129, 139)	<0.001
Glucose (mg/dl)	141 (105, 198)	156 (113, 214)	114 (92, 144)	<0.001
Creatinine (mg/dl)	1.10 (0.80, 1.90)	1.00 (0.75, 1.60)	1.65 (1.00, 2.70)	<0.001
Direct bilirubin (mg/dl)	2.5 (1.2, 5.4)	2.1 (1.1, 4.3)	4.4 (2.2, 11.8)	<0.001
Fibrinogen (mg/dl)	181 (141, 228)	184 (147, 228)	169 (123, 231)	0.046
Lactate	2.40 (1.70, 3.50)	2.17 (1.50, 2.93)	3.50 (2.50, 4.50)	<0.001
ALT(IU/L)	60 (27, 298)	107 (28, 408)	43 (27, 81)	<0.001
ALP(IU/L)	94 (66, 149)	87 (64, 134)	114 (82, 183)	<0.001
AST(IU/L)	141 (60, 579)	205 (63, 731)	100 (59, 189)	<0.001
WBC (10^9/L)	9(5, 13)	8 (5, 12)	11 (8, 19)	<0.001
Platelet(10^9/L)	94 (60, 138)	93 (61, 131)	105 (56, 153)	0.613
Hemoglobin(10^9/L)	9.50 (8.15, 11.30)	9.60 (8.30, 11.40)	9.20 (7.70, 10.70)	0.060
INR	1.70 (1.40, 2.25)	1.60 (1.30, 2.00)	2.10 (1.80, 2.80)	<0.001
PT(s)	19 (16, 24)	18 (15, 22)	23 (19, 29)	<0.001
APTT(s)	39 (33, 50)	38 (32, 47)	42 (36, 57)	<0.001
Albumin(mg/dl)	2.90 (2.50, 3.20)	2.90 (2.50, 3.20)	2.70 (2.40, 3.20)	0.093
Chloride (m Eq/l)	103 (98, 106)	104 (100, 106)	100 (94, 105)	<0.001
DBAR	0.90 (0.45, 2.13)	0.75 (0.38, 1.56)	1.63 (0.83, 4.29)	<0.001
MELD	20.0 (13.0, 28.0)	18.0 (12.0, 25.0)	28.0 (21.0, 34.0)	<0.001
**Comorbidities**				
Diabetes, n (%)	127 (25.0%)	101 (26.9%)	26 (19.4%)	0.063
Renal disease, n (%)	74 (14.5%)	48 (12.8%)	26 (19.4%)	0.063
AKI, n (%)	432 (84.9%)	308 (82.1%)	124 (92.5%)	0.046
Sepsis, n (%)	411 (80.7%)	296 (78.9%)	115 (85.8%)	0.083
SIRS	457 (89.8%)	333 (88.8%)	124 (92.5%)	0.220
Hepatorenal syndrome, n (%)	80 (15.7%)	45 (12.0%)	35 (26.1%)	<0.001
Variceal bleeding, n (%)	17 (3.3%)	14 (3.7%)	3 (2.2%)	0.578
Ascites, n (%)	289 (56.8%)	196 (52.3%)	93 (69.4%)	<0.001
Spontaneous bacterial peritonitis, n (%)	68 (13.4%)	40 (10.7%)	28 (20.9%)	0.003
**Treatment**				
Vasoactive, n (%)	240 (47.2%)	144 (38.4%)	96 (71.6%)	<0.001
Ventilator, n (%)	417 (81.9%)	310 (82.7%)	107 (79.9%)	0.467
CRRT, n (%)	76 (14.9%)	40 (10.7%)	36 (26.9%)	<0.001
**Group**				
DBAR (<4), n (%)	444 (87.2%)	348 (92.8%)	96 (71.6%)	<0.001
DBAR (≥4), n (%)	65 (12.8%)	27 (7.2%)	38 (28.4%)	

Ca: calcium; BUN: Blood Urea Nitrogen; AST: aspartate aminotransferase; ALT: alanine aminotransferase; WBC: white blood cell; INR: international normalized ratio; PT: prothrombin time; APTT: Activated Partial Thromboplastin Time; ALP: Alkaline Phosphatase; LDH: Lactate Dehydrogenase; SIRS: Systemic Inflammatory Response Syndrome; MELD: Model for End-Stage Liver Disease; CRRT: continuous renal replacement therapy; AKI: acute kidney injury; DBAR: Direct Bilirubin-to-Albumin Ratio.

### 2.4 Statistical analyses

Continuous variables were reported as mean ± SD or median (IQR), and categorical variables as counts (%). Baseline features were compared using Student’s t test, ANOVA, chi-square, or Fisher’s exact test. Variables with >20% missing values were excluded, while those with <20% were imputed using random forest. Univariate Cox regression analysis was conducted to identify significant variables, which were then included in the multivariate Cox regression model based on clinical relevance and statistical significance. The DBAR cut-off (DBAR < 4 vs. ≥ 4) was determined using X-tile software (version 3.6.1, Yale University), with adjusted covariates to assess survival differences. KM curves and log-rank tests were used to evaluate survival probabilities. ROC analysis assessed the predictive performance of DBAR, direct bilirubin, albumin, and MELD scores, reporting sensitivity, specificity, and Area Under the Curve (AUC). In order to thoroughly evaluate the impact of various factors on the prediction of cirrhosis outcomes, we utilized the Boruta Feature Selection method, a highly regarded approach in the field of machine learning, to pinpoint the most critical predictive variables. RCS analysis explored potential nonlinear associations between DBAR and prognosis. Subgroup analyses examined the impact of covariates (age, sex, race, sepsis, SBP, ascites, HRS, vasoactive treatment) on the DBAR-mortality relationship. Analyses were performed using R (version 4.2.2, R Foundation).

## 3 Results

### 3.1 Baseline clinical characteristics

A total of 509 patients were included in this study, with a mean age of 59 years and a predominance of male (69.7%) and Caucasian (65.6%) individuals. Comparative analysis between the mortality and survival groups demonstrated that patients in the mortality group exhibited significant elevations in multiple clinical parameters, including heart rate, respiratory rate, systolic blood pressure, anion gap, BUN, potassium, WBC, direct bilirubin, creatinine and INR. Furthermore, the mortality group had substantially higher MELD scores (28.0 vs. 18.0). Additionally, the mortality group showed a higher prevalence of HRS (26.1% vs. 12%), ascites (69.4% vs. 52.3%), SBP (20.9% vs. 10.7%), AKI (92.5% vs. 82.1%), CRRT (26.9% vs. 10.7%), and vasopressor use (71.6% vs. 38.4%). Stratification based on DBAR revealed a stark contrast in survival rates between the two groups (58.4% vs. 21.6%). However, no statistically significant differences were observed in baseline demographics or clinical features, including age, diabetes, sepsis, and ventilator use (P ≥ 0.05). The baseline characteristics of patients in both groups are detailed in Table 1.

### 3.2 Both univariate and multivariate Cox regression analyses were performed to identify clinical predictors of mortality during the 28-day follow-up

In the univariate Cox proportional hazards analysis, non-surviving patients exhibited significantly higher levels of age, DBAR, BUN, potassium, creatinine, lactate, WBC, INR, SBP, as well as increased utilization of CRRT and vasoactive agents. Additionally, a higher prevalence of SBP, hepatorenal syndrome, and ascites was observed in this group. Conversely, lower levels of sodium, chloride, ALT, and AST were noted in non-survivors.

In the multivariate Cox proportional hazards model, several variables were independently associated with 28-day mortality, including DBAR (HR = 1.16, 95% CI 1.10–1.24; p < 0.001), Age (HR = 1.03, 95% CI 1.02–1.05; p < 0.001), BUN (HR = 1.01, 95% CI 1.00–1.01; p = 0.006), Lactate (HR = 1.21, 95% CI 1.15–1.27; p < 0.001), ALT (HR = 1.00, 95% CI 1.00–1.00; p < 0.001), INR (HR = 1.17, 95% CI 1.01–1.37; p = 0.041), and vasoactive agent use (HR = 2.56, 95% CI 1.72–3.81; p < 0.001). Detailed results are presented in Table 2.

**Table 2:** Cox regression analyses of clinical parameters associated with 28-day mortality.

Variables	Univariate analysis			Multivariate analysis		
	HR	95% CI	p-value	HR	95% CI	p-value
Age(year)	1.02	1.00, 1.04	0.016	1.03	1.02, 1.05	<0.001
Gender, n (%)						
Male						
Female	1.34	0.94, 1.91	0.104			
Race, n (%)						
Other	–	–	–			
White	0.73	0.52,1.03	0.072			
Respiratory Rate(beats/min)	1.06	1.04, 1.09	<0.001			
Heart Rate(beats/min)	1.01	1.00, 1.02	0.003			
Temperature (°C)	0.65	0.57, 0.74	<0.001			
DBAR	1.18	1.13, 1.24	<0.001	1.16	1.10, 1.24	<0.001
BUN (mg/dl)	1.02	1.01, 1.02	<0.001	1.01	1.00, 1.01	0.006
Bicarbonate (m Eq/l)	0.90	0.86, 0.93	<0.001			
Potassium (m Eq/l)	1.35	1.13, 1.60	<0.001			
Sodium (m Eq/l)	0.95	0.93, 0.97	<0.001			
Creatinine (mg/dl)	1.16	1.08, 1.24	<0.001			
Fibrinogen(mg/dl)	1.00	1.00, 1.00	0.588			
Lactate (mmol/L)	1.21	1.17, 1.26	<0.001	1.21	1.15, 1.27	<0.001
ALT(IU/L)	1.00	1.00, 1.00	<0.001	1.00	1.00, 1.00	<0.001
AST(IU/L)	1.00	1.00, 1.00	0.012			
WBC (10^9/L)	1.06	1.04, 1.07	<0.001			
Platelet(10^9/L)	1.00	1.00, 1.00	0.454			
Hemoglobin(10^9/L)	0.94	0.87, 1.02	0.135			
INR	1.67	1.47, 1.90	<0.001	1.17	1.01, 1.37	0.041
Chloride (m Eq/l)	0.97	0.95, 0.99	<0.001			
AKI, n (%)	2.47	1.29, 4.70	0.006			
Sepsis, n (%)	1.55	0.96, 2.53	0.075			
Spontaneous bacterial peritonitis, n (%)	1.80	1.19, 2.73	0.006			
Hepatorenal syndrome, n (%)	2.12	1.44, 3.12	<0.001			
SIRS, n (%)	1.52	0.80, 2.89	0.205			
Variceal bleeding, n (%)	0.60	0.19, 1.88	0.377			
Ascites, n (%)	1.82	1.26, 2.62	0.001			
Vasoactive, n (%)	3.42	2.35, 4.98	<0.001	2.56	1.72, 3.81	<0.001
Ventilator, n (%)	0.87	0.57, 1.32	0.502			
CRRT, n (%)	2.51	1.71, 3.68	<0.001			

Ca, calcium; BUN, Blood Urea Nitrogen; AST, aspartate aminotransferase; ALT, alanine aminotransferase; WBC, white blood cell; INR, international normalized ratio; LDH: Lactate Dehydrogenase; SIRS: Systemic Inflammatory Response Syndrome; CRRT, continuous renal replacement therapy; AKI, acute kidney injury; DBAR: Direct Bilirubin-to-Albumin Ratio; HR: Hazard Ratio; 95 CI: 95% Confidence Interval.

### 3.3 Relationship between DBAR and 28-day mortality

In the multivariate Cox regression analysis, variables were selected for inclusion based on their statistical significance in univariate analysis or their established clinical relevance to ensure accurate model adjustment. In the initial model (Model 1), the HR for the association between DBAR and 28-day mortality was 1.21 (95% CI 1.15–1.26; p < 0.001). Model 2, which incorporated additional clinically pertinent covariates, demonstrated a slightly attenuated HR for DBAR of 1.15 (95% CI 1.09–1.21; p < 0.001). Extending Model 2, Model 3 further reinforced the independent prognostic value of DBAR, with an HR of 1.17 (95% CI 1.09–1.26; p < 0.001). These findings robustly establish DBAR as a significant and independent predictor of 28-day mortality among critically ill cirrhotic patients with cirrhosis.

To investigate the differential impact of DBAR levels, we utilized X-tile software to stratify patients according to 28-day mortality. The cohort was categorized into two distinct groups: a high-risk group (DBAR ≥ 4) and a low-risk group (DBAR < 4). In Model 1, which adjusted for age, sex, and race, the high-risk group exhibited a significantly elevated mortality rate, with a HR of 4.82 (95% CI 3.22–7.23; p < 0.001). Further adjustments in Model 2 and Model 3 consistently affirmed the heightened mortality risk in the high-risk group, highlighting a robust and independent association between DBAR ≥ 4 and mortality. Specifically, Model 2 yielded an HR of 3.22 (95% CI 2.10–5.24; p < 0.001), and Model 3 produced an HR of 3.05 (95% CI 1.87–4.97; p < 0.001). Comprehensive results are summarized in Table 3.

**Table 3:** Association between DBAR and mortality in patients with cirrhosis.

Variables	Model 1			Model 2			Model 3		
	HR	95% CI	p-value	HR	95% CI	p-value	HR	95% CI	p-value
DBAR	1.21	1.15, 1.26	<0.001	1.15	1.09, 1.21	<0.001	1.17	1.09, 1.26	<0.001
DBAR Group									
DBAR<4	1(Reference)		1(Reference)	1(Reference)		1(Reference)	1(Reference)		1(Reference)
DBAR≥4	4.82	3.22, 7.23	<0.001	3.32	2.10, 5.24	<0.001	3.05	1.87, 4.97	<0.001

Model 1: adjusted for age, gender, and race;

Model 2: adjusted for age, gender, race, BUN, creatinine, and SIRS;

Model 3: adjusted for age, gender, race, BUN, creatinine, SIRS, potassium, sodium, lactate, WBC, platelet, INR, Sepsis, AKI, SBP, HRS, Variceal bleeding, Ascites, Vasoactive, MV, and CRRT.

### 3.4 Analysis of Kaplan-Meier and ROC curves

**Fig 2:**
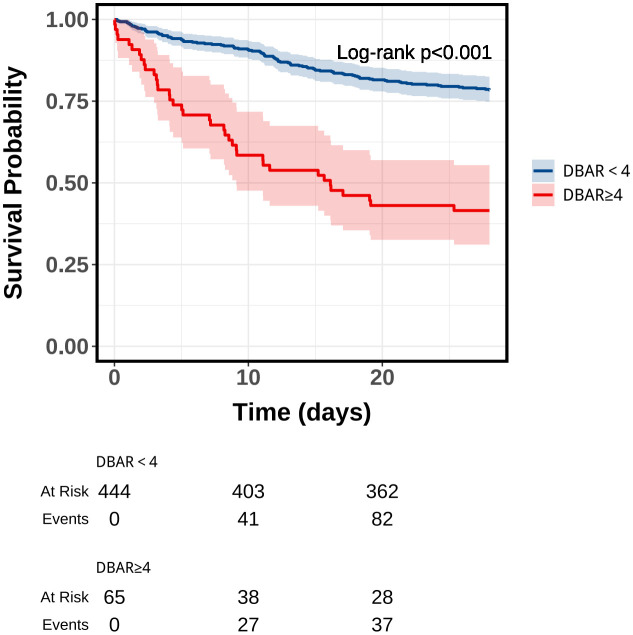
Kaplan-Meier survival analysis curves for all-cause mortality in patients with cirrhosis at 28-d of hospital admission.

**Fig 3:**
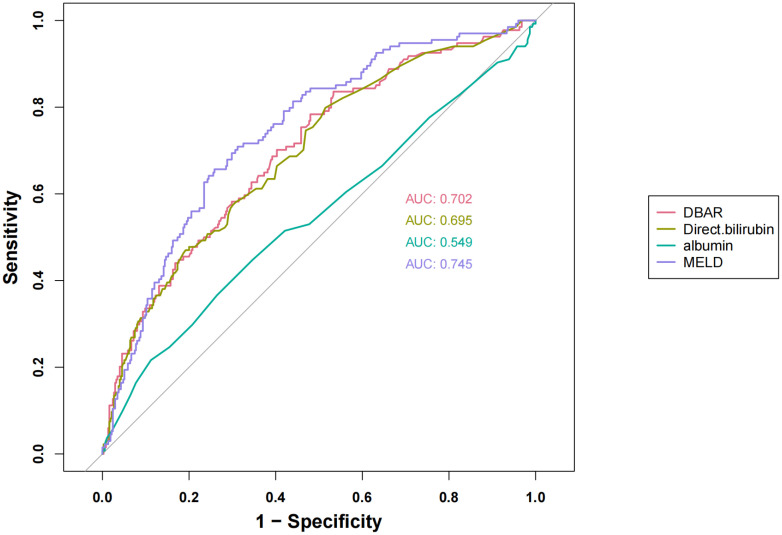
DBAR and 28-day all-cause mortality: ROC curves.

A KM survival curve analysis demonstrated a significantly higher mortality rate at the 28-day follow-up for patients with a DBAR ≥ 4 compared to those with a DBAR < 4 (56.9% vs. 18.4%, respectively; Fig 2). The AUC for DBAR in predicting short-term mortality among this patient population was 0.702 (95% CI: 0.650–0.753), which, while marginally lower than the more complex MELD score, still reflects moderate to good discriminative performance. These findings suggest that DBAR serves as a reliable prognostic tool, exhibiting predictive accuracy comparable to the established and widely validated MELD score within the same patient population. Furthermore, DBAR exhibited consistent predictive capacity for in-hospital mortality (AUC = 0.720) and 90-day mortality (AUC = 0.676). Comprehensive data are presented in Fig 3 and Table 4.

**Table 4:** Details of ROC curves shown in Fig 3.

Variable	AUC	95%CI	Threshold	Sensitivity	Specificity
DBAR	0.702	0.650 - 0.753	0.777	0.784	0.520
Direct bilirubin	0.695	0.643 - 0.747	2.000	0.799	0.485
Albumin	0.549	0.489 - 0.609	2.200	0.216	0.888
MELD	0.744	0.697 - 0.792	22.619	0.709	0.689

### 3.5 DBAR feature importance in cirrhosis prognosis (Boruta analysis)

**Fig 4:**
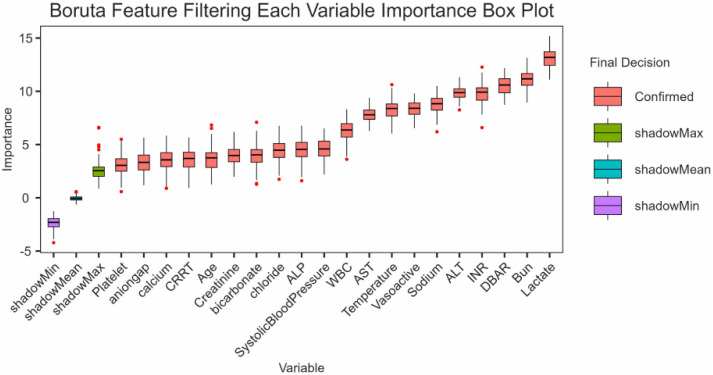
DBAR and cirrhosis prognosis: Boruta feature importance analysis.

To comprehensively assess the significance of variables in predicting cirrhosis prognosis, we employed Boruta Feature Selection, a widely recognized machine learning algorithm, to identify key predictors. The analysis highlighted lactate, blood urea nitrogen, and DBAR as the top three most influential factors. This result further substantiates the robust relationship between DBAR and cirrhosis prognosis. A detailed representation of these findings is provided in Fig 4.

### 3.6 RCS analysis of DBAR in relation to cirrhosis prognosis

**Fig 5:**
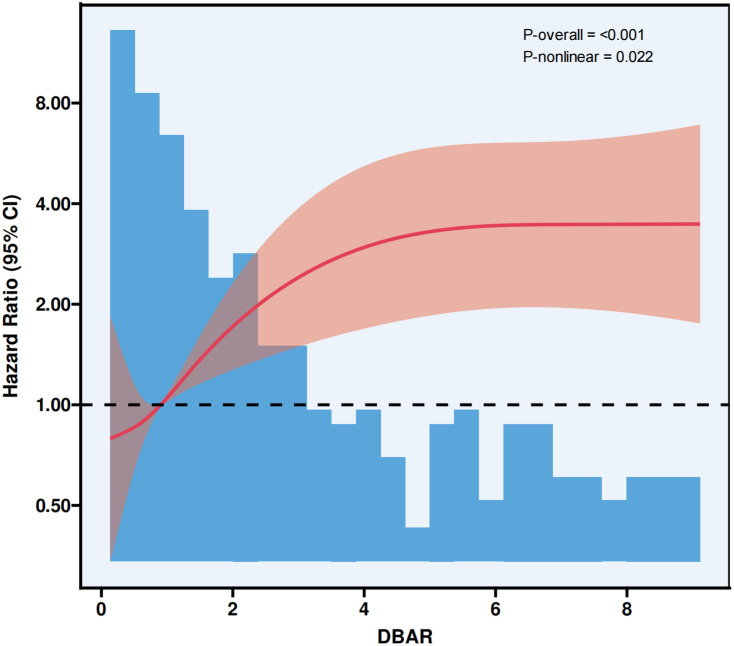
DBAR and survival: RCS analysis.

Our study utilized RCS analysis to uncover a notable nonlinear association between the DBAR and cirrhosis prognosis, with P-values for nonlinearity and overall significance of 0.022 and <0.001, respectively. This finding indicates that higher DBAR levels are significantly associated with an increased risk of adverse clinical outcomes. For a comprehensive visualization of these results, please refer to Fig 5.

### 3.7 Clinical outcomes by DBAR subgroup in cirrhosis

**Fig 6:**
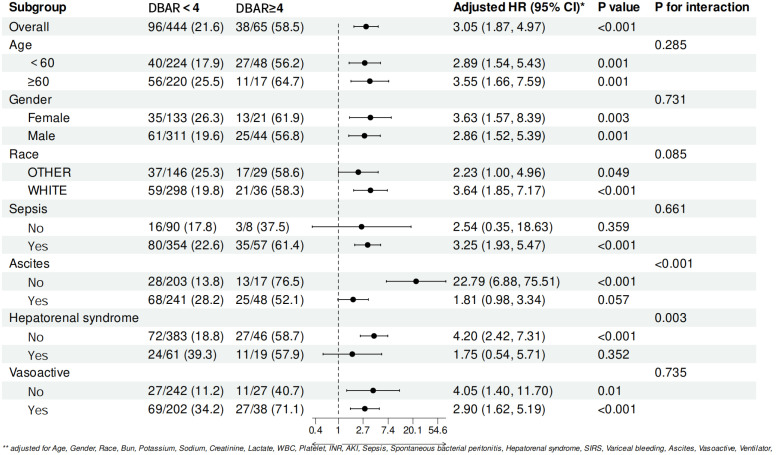
Forest plots showing subgroup effects of DBAR on 28-day all-cause mortality in cirrhosis.

In the subgroup analyses, the influence of demographic and clinical variables—including age, sex, race and sepsis—on patient outcomes was evaluated. No statistically significant interactions between these variables and the DBAR were observed. However, the predictive performance of the DBAR exhibited variability across subgroups defined by the presence of ascites and hepatorenal syndrome, indicating that its prognostic utility may be contingent upon these specific clinical conditions. Notably, the presence of ascites emerged as a potential explanatory factor for the differential predictive accuracy observed. The comprehensive results of these analyses are presented in Fig 6.

### 3.8 External validation using eICU-CRD cohort

To validate the robustness of our findings, we further analyzed an independent validation cohort from the eICU Collaborative Research Database (eICU-CRD), a large multicenter critical care database comprising over 200,000 ICU admissions across the United States. Consistent with the results from the MIMIC-IV cohort, DBAR remained an independent predictor of short-term mortality in the eICU-CRD population after adjustment in multivariable Cox regression models. RCS analysis confirmed a nonlinear dose–response association between DBAR and short-term mortality risk. Moreover, ROC curve analysis demonstrated good discriminative ability, with an AUC of approximately 0.71. These findings further corroborate the robustness and generalizability of DBAR as a prognostic biomarker in critically ill cirrhotic patients.

Importantly, the prognostic value of DBAR was further confirmed in an independent external validation cohort (eICU-CRD), underscoring its robustness and generalizability across diverse ICU populations.

The restricted cubic spline analysis and ROC analysis of the validation cohort are provided as S1 and S2 Figs. The detailed results of the multivariable Cox regression models are presented in S1 Table.

## 4 Discussion

Cirrhosis, a chronic liver disease with significant global prevalence, is closely linked to elevated mortality rates, especially in individuals with cirrhosis [19]. Within ICU settings, prognostic scoring systems such as the MELD and the Child-Pugh score are standard tools for assessing the prognosis of this population. Emerging research has identified several specific ratios—including the international normalized ratio to albumin ratio [20], lactate to albumin ratio [21], and neutrophil to albumin ratio [22]—as independent risk factors for cirrhosis, thereby improving prognostic accuracy. Nevertheless, there remains a pressing clinical need for prognostic indicators that are both simplified and more easily interpretable to enhance practical utility.

In this study, univariate and multivariate prognostic analyses were performed on a cohort of critically ill cirrhotic patients, revealing that the DBAR serves as an independent predictor of short-term all-cause mortality. Patients were categorized into high-risk (DBAR ≥4) and low-risk (DBAR <4) groups based on DBAR thresholds. KM survival analysis demonstrated a persistently significant prognostic disparity in the high-risk group, even after adjusting for multiple covariates (HR: 3.05, p < 0.001). Both DBAR and the MELD score exhibited moderate discriminative capacity in predicting 28-day mortality, with consistent predictive accuracy for in-hospital (ROC: 0.720) and 90-day mortality (ROC: 0.676). These findings underscore the potential clinical significance of DBAR in evaluating both short- and medium-to-long-term prognoses in patients with advanced cirrhosis admitted to the ICU. RCS analysis revealed a significant nonlinear association between DBAR and cirrhosis. Hazard ratios increased progressively with higher DBAR values. Subgroup analysis revealed variability in the predictive efficacy of DBAR among patients with ascites and HRS. This variability can be attributed to two primary factors: First, severe ascites is frequently associated with renal sodium retention and diminished effective circulating blood volume, which may exacerbate renal dysfunction, disrupt albumin metabolism, and impair bilirubin clearance. Second, the imperative for aggressive interventions, such as paracentesis and albumin supplementation, in these patients may introduce interaction effects that influence prognosis. Importantly, the prognostic significance of DBAR was further confirmed in an independent external validation cohort (eICU-CRD), which underscores the robustness and generalizability of our findings across diverse ICU populations. This study elucidates the potential role of DBAR as a prognostic biomarker in critically ill cirrhotic patients, while highlighting the complex interplay of pathophysiological mechanisms in advanced liver disease.

The Albumin-Bilirubin (ALBI) score, primarily utilized for grading liver cancer prognosis, has also found application in assessing critically ill cirrhotic patients [14]. In a case-control study involving 204 patients with liver failure, Li et al. [16] demonstrated that the ratio of indirect bilirubin to albumin is significantly associated with the prognosis of hepatic encephalopathy (HR: 1.626, P < 0.001), underscoring the prognostic relevance of the bilirubin-to-albumin ratio in cirrhosis-related complications. While direct bilirubin, a marker of hepatic metabolic function, has been strongly linked to cirrhosis prognosis, indirect bilirubin—often elevated due to hemolytic reactions—plays a less critical role in cirrhotic patient survival compared to intrahepatic cholestasis and impaired hepatic clearance [17]. This suggests that direct bilirubin levels are more strongly predictive of poor prognosis in cirrhosis than indirect bilirubin (OR:1.373). Furthermore, a separate study highlighted the prognostic utility of the direct bilirubin to total bilirubin ratio in liver failure [23].

Serum albumin, exclusively synthesized by the liver, serves as a key indicator of hepatic synthetic function and nutritional status [12]. Hypoalbuminemia, commonly observed in cirrhotic patients [24,25], is strongly associated with increased mortality [3] and can exacerbate complications such as ascites and edema, thereby worsening the clinical course of the disease [26,27]. This condition may arise from inflammation-induced extravascular albumin loss, impaired hepatic synthesis, or malnutrition, all of which heighten infection risk and may signal impending organ failure [28,29]. Low albumin levels have also been linked to poor outcomes in acutely ill patients [30,31], further emphasizing its prognostic significance. Consequently, hypoalbuminemia is closely correlated with disease severity and adverse prognosis in cirrhosis. To leverage the complementary prognostic value of bilirubin and albumin, we utilized an inverse variation mechanism to mitigate the impact of individual indicators, specifically through the direct bilirubin to albumin ratio.

The complexity of certain ICU scoring systems can hinder their practical application in the routine management of critically ill cirrhotic patients. Widely used scoring systems, such as MELD and Child-Pugh, may be compromised by the subjectivity associated with evaluating hepatic encephalopathy. In contrast, the DBAR offers simplicity without compromising accuracy. Our study demonstrates that elevated DBAR levels are strongly associated with the severity of cirrhosis and poor prognosis, establishing it as a reliable indicator for assessing patient condition and outcomes. By integrating information on liver function, inflammation, and nutritional status, DBAR provides more comprehensive insights compared to isolated evaluations of direct bilirubin or albumin. An elevated DBAR reflects increased direct bilirubin levels and decreased albumin levels—two critical biomarkers. Elevated direct bilirubin suggests impaired hepatic function and disease severity, while reduced albumin levels may indicate impaired liver synthesis, albumin loss, inflammatory consumption, or malnutrition, all of which are linked to poor prognosis. DBAR effectively leverages the combined prognostic value of direct bilirubin and albumin in severe cirrhosis. Clinicians can use DBAR values to guide treatment decisions, employing standard therapy for patients below the threshold and opting for more intensive interventions for those above it. This stratification enables the efficient identification of severe cases and optimizes the allocation of medical resources, thereby improving the precision and effectiveness of clinical care. Furthermore, since direct bilirubin and albumin are routinely measured in standard blood tests, calculating DBAR does not require additional laboratory work, minimizing the financial burden on patients. Overall, DBAR emerges as a valuable prognostic tool for assessing severe cirrhosis by synergistically combining direct bilirubin and albumin levels. Its ease of use, cost-effectiveness, and robust predictive capacity make it a practical and reliable indicator in clinical practice.

In this study, we utilized data from publicly available databases to investigate prognostic factors associated with the disease. The primary strength of this approach is the access to a comprehensive real-world dataset; however, several significant limitations must be addressed. First, the inclusion of patients without severity stratification restricts our capacity to evaluate potential differences across populations with varying disease severity. Second, the absence of detailed etiology data for cirrhosis in critically ill cirrhotic patients further impedes our analysis of variations among patient groups with different underlying causes. Additionally, we were unable to determine the precise cause of death for all patients; thus, the observed mortality rate in critically ill patient

individuals may be influenced by multiple factors, and our analysis treats this as all-cause mortality. Furthermore, the study population exclusively comprised patients admitted to the ICU, a characteristic that may introduce selection bias and limit the generalizability of our conclusions.

## 5 Conclusion

DBAR emerges as an independent prognostic marker for mortality among critically ill cirrhotic patients and represents a readily accessible laboratory parameter for identifying this high-risk population. However, additional large-scale, prospective, multicenter studies are warranted to comprehensively evaluate and validate its clinical applicability.

## Supporting information

S1 File The compiled database used for all analyses in this study. (RAR)

S1 Table Association between DBAR and mortality in the eICU-CRD validation cohort. (DOCX)

S1 Fig DBAR and mortality in the eICU-CRD cohort (RCS analysis). (TIF)

S2 Fig DBAR and mortality in the eICU-CRD cohort (ROC analysis). (TIF)

## References

[1] Bernardi M, Moreau R, Angeli P, Schnabl B, Arroyo V. Mechanisms of decompensation and organ failure in cirrhosis: from peripheral arterial vasodilation to systemic inflammation hypothesis. J Hepatol. 2015;63(5):1272–84. doi: 10.1016/j.jhep.2015.07.004 26192220

[2] Zhou W-C, Zhang Q-B, Qiao L. Pathogenesis of liver cirrhosis. World J Gastroenterol. 2014;20(23):7312–24. doi: 10.3748/wjg.v20.i23.7312 24966602 PMC4064077

[3] D’Amico G, Garcia-Tsao G, Pagliaro L. Natural history and prognostic indicators of survival in cirrhosis: a systematic review of 118 studies. J Hepatol. 2006;44(1):217–31. doi: 10.1016/j.jhep.2005.10.013 16298014

[4] Garcia-Tsao G, Abraldes JG, Berzigotti A, Bosch J. Portal hypertensive bleeding in cirrhosis: risk stratification, diagnosis, and management: 2016 practice guidance by the American Association for the study of liver diseases. Hepatology. 2017;65(1):310–35. doi: 10.1002/hep.28906 27786365

[5] Olson JC, Wendon JA, Kramer DJ, Arroyo V, Jalan R, Garcia-Tsao G, et al. Intensive care of the patient with cirrhosis. Hepatology. 2011;54(5):1864–72. doi: 10.1002/hep.24622 21898477

[6] Tu K-H, Jenq C-C, Tsai M-H, Hsu H-H, Chang M-Y, Tian Y-C, et al. Outcome scoring systems for short-term prognosis in critically ill cirrhotic patients. Shock. 2011;36(5):445–50. doi: 10.1097/SHK.0b013e31822fb7e2 21841535

[7] Gildea TR, Cook WC, Nelson DR, Aggarwal A, Carey W, Younossi ZM, et al. Predictors of long-term mortality in patients with cirrhosis of the liver admitted to a medical ICU. Chest. 2004;126(5):1598–603. doi: 10.1378/chest.126.5.1598 15539733

[8] Bahirwani R, Ghabril M, Forde KA, Chatrath H, Wolf KM, Uribe L, et al. Factors that predict short-term intensive care unit mortality in patients with cirrhosis. Clin Gastroenterol Hepatol. 2013;11(9):1194-1200.e2. doi: 10.1016/j.cgh.2013.03.035 23602820 PMC3873858

[9] Albers I, Hartmann H, Bircher J, Creutzfeldt W. Superiority of the Child-Pugh classification to quantitative liver function tests for assessing prognosis of liver cirrhosis. Scand J Gastroenterol. 1989;24(3):269–76. doi: 10.3109/00365528909093045 2734585

[10] Kamath PS, Wiesner RH, Malinchoc M, Kremers W, Therneau TM, Kosberg CL, et al. A model to predict survival in patients with end-stage liver disease. Hepatology. 2001;33(2):464–70. doi: 10.1053/jhep.2001.22172 11172350

[11] Ramírez-Mejía MM, Castillo-Castañeda SM, Pal SC, Qi X, Méndez-Sánchez N. The multifaceted role of bilirubin in liver disease: a literature review. J Clin Transl Hepatol. 2024;12(11):939–48. doi: 10.14218/JCTH.2024.00156 39544246 PMC11557368

[12] Akirov A, Masri-Iraqi H, Atamna A, Shimon I. Low albumin levels are associated with mortality risk in hospitalized patients. Am J Med. 2017;130(12):1465.e11-1465.e19. doi: 10.1016/j.amjmed.2017.07.020 28803138

[13] Santol J, Kim S, Gregory LA, Baumgartner R, Murtha-Lemekhova A, Birgin E, et al. An APRI+ALBI-based multivariable model as a preoperative predictor for posthepatectomy liver failure. Ann Surg. 2025;281(5):861–71. doi: 10.1097/SLA.0000000000006127 37860868 PMC11974630

[14] Deng M, Ng SWY, Cheung ST, Chong CCN. Clinical application of Albumin-Bilirubin (ALBI) score: the current status. Surgeon. 2020;18(3):178–86. doi: 10.1016/j.surge.2019.09.002 31601470

[15] Ho S-Y, Liu P-H, Hsu C-Y, Ko C-C, Huang Y-H, Su C-W, et al. ALBI grade in dialysis patients with hepatocellular carcinoma: prognostic impact and staging strategy. J Gastrointest Oncol. 2021;12(2):722–34. doi: 10.21037/jgo-20-332 34012661 PMC8107592

[16] Li Y, Liu H, Chen K, Wu X, Wu J, Yang Z, et al. Pathological significance and prognostic roles of indirect Bilirubin/Albumin ratio in hepatic encephalopathy. Front Med (Lausanne). 2021;8:706407. doi: 10.3389/fmed.2021.706407 34527681 PMC8435674

[17] Lee HA, Jung JY, Lee Y-S, Jung YK, Kim JH, An H, et al. Direct bilirubin is more valuable than total bilirubin for predicting prognosis in patients with liver cirrhosis. Gut Liver. 2021;15(4):599–605. doi: 10.5009/gnl20171 33293481 PMC8283287

[18] Johnson AEW, Bulgarelli L, Shen L, Gayles A, Shammout A, Horng S, et al. MIMIC-IV, a freely accessible electronic health record dataset. Sci Data. 2023;10(1):1. doi: 10.1038/s41597-022-01899-x 36596836 PMC9810617

[19] Huang DQ, Terrault NA, Tacke F, Gluud LL, Arrese M, Bugianesi E, et al. Global epidemiology of cirrhosis - aetiology, trends and predictions. Nat Rev Gastroenterol Hepatol. 2023;20(6):388–98. doi: 10.1038/s41575-023-00759-2 36977794 PMC10043867

[20] Zhou Y-J, Zheng J-N, Zhou Y-F, Han Y-J, Zou T-T, Liu W-Y, et al. Development of a prognostic nomogram for cirrhotic patients with upper gastrointestinal bleeding. Eur J Gastroenterol Hepatol. 2017;29(10):1166–73. doi: 10.1097/MEG.0000000000000943 28746121

[21] Krispin I, Mahamid M, Goldin E, Fteiha B. Elevated lactate/albumin ratio as a novel predictor of in-hospital mortality in hospitalized cirrhotics. Ann Hepatol. 2023;28(3):100897. doi: 10.1016/j.aohep.2023.100897 36632976

[22] Yao J, Xu X, Gong K, Tu H, Xu Z, Ye S, et al. Prognostic value of neutrophil count to albumin ratio in patients with decompensated cirrhosis. Sci Rep. 2023;13(1):20759. doi: 10.1038/s41598-023-44842-9 38007536 PMC10676395

[23] Ma Y, Du L, Zhou S, Bai L, Tang H. Association of direct bilirubin to total bilirubin ratio with 90-day mortality in patients with acute-on-chronic liver failure. Front Med (Lausanne). 2023;10:1286510. doi: 10.3389/fmed.2023.1286510 38020137 PMC10666058

[24] Bernardi M, Ricci CS, Zaccherini G. Role of human albumin in the management of complications of liver cirrhosis. J Clin Exp Hepatol. 2014;4(4):302–11. doi: 10.1016/j.jceh.2014.08.007 25755577 PMC4298636

[25] Romanelli R-G, La Villa G, Barletta G, Vizzutti F, Lanini F, Arena U, et al. Long-term albumin infusion improves survival in patients with cirrhosis and ascites: an unblinded randomized trial. World J Gastroenterol. 2006;12(9):1403–7. doi: 10.3748/wjg.v12.i9.1403 16552809 PMC4124318

[26] Rossiter N. Levelling up: prioritisation of global health. Eur J Orthop Surg Traumatol. 2023;33(3):559–63. doi: 10.1007/s00590-022-03394-w 36173480 PMC9521009

[27] Carvalho JR, Verdelho Machado M. New insights about albumin and liver disease. Ann Hepatol. 2018;17(4):547–60. doi: 10.5604/01.3001.0012.0916 29893696

[28] Artigas A, Wernerman J, Arroyo V, Vincent J-L, Levy M. Role of albumin in diseases associated with severe systemic inflammation: pathophysiologic and clinical evidence in sepsis and in decompensated cirrhosis. J Crit Care. 2016;33:62–70. doi: 10.1016/j.jcrc.2015.12.019 26831575

[29] Wiedermann CJ. Hypoalbuminemia as surrogate and culprit of infections. Int J Mol Sci. 2021;22(9):4496. doi: 10.3390/ijms22094496 33925831 PMC8123513

[30] Chaudhuri D, Sasaki K, Karkar A, Sharif S, Lewis K, Mammen MJ, et al. Corticosteroids in COVID-19 and non-COVID-19 ARDS: a systematic review and meta-analysis. Intensive Care Med. 2021;47(5):521–37. doi: 10.1007/s00134-021-06394-2 33876268 PMC8054852

[31] Dubois M-J, Orellana-Jimenez C, Melot C, De Backer D, Berre J, Leeman M, et al. Albumin administration improves organ function in critically ill hypoalbuminemic patients: a prospective, randomized, controlled, pilot study. Crit Care Med. 2006;34(10):2536–40. doi: 10.1097/01.CCM.0000239119.57544.0C 16915107

