# Supplementary material for: The prognostic value of the direct bilirubin to albumin ratio in critically ill patients with cirrhosis: Insights from MIMIC-IV database
Source: PLoS One. 2025 Oct 13;20(10):e0334591. doi: 10.1371/journal.pone.0334591 (PMC12517503; doi:10.1371/journal.pone.0334591)
Supplement: S1 Table — (DOCX) [file pone.0334591.s002.docx]

**Supplementary Table 1:** Association between DBAR and mortality in the eICU-CRD validation cohort.

| **Variables** | **Model 1** | | | **Model 2** | | | **Model 3** | | |
| --- | --- | --- | --- | --- | --- | --- | --- | --- | --- |
|  | **HR** | **95% CI** | **p-value** | **HR** | **95% CI** | **p-value** | **HR** | **95% CI** | **p-value** |
| DBAR (continuous) | 1.13 | 1.08, 1.19 | <0.001 | 1.12 | 1.06, 1.18 | <0.001 | 1.07 | 1.00, 1.15 | 0.038 |
| DBAR |  |  |  |  |  |  |  |  |  |
| Q1 [0,0.305) | 1(Reference) | | | 1(Reference) | | | 1(Reference) | | |
| Q2 [0.305,0.821) | 2.39 | 1.07, 5.35 | 0.034 | 2.48 | 1.10, 5.58 | 0.028 | 3.02 | 1.29, 7.12 | 0.011 |
| Q3 [0.821,2.69) | 5.36 | 2.57, 11.18 | <0.001 | 5.25 | 2.51, 10.98 | <0.001 | 4.36 | 2.00, 9.50 | <0.001 |
| Q4 [2.69,17.6] | 7.89 | 3.77, 16.52 | <0.001 | 7.36 | 3.51, 15.46 | <0.001 | 5.12 | 2.21, 11.87 | <0.001 |
| P for trend |  |  | <0.001 |  |  | <0.001 |  |  | <0.001 |

Model 1: adjusted for age, gender, and race;

Model 2: adjusted for age, gender, race, bun, creatinine;

Model 3: adjusted for age, gender, race, bun, creatinine, potassium, sodium, lactate, WBC, platelet,

INR, Sepsis, AKI, Spontaneous bacterial peritonitis, Hepatorenal syndrome, Variceal

bleeding, Ascites, Vasoactive, Ventilator, and CRRT
