# Supplementary figures and images for: The prognostic value of the direct bilirubin to albumin ratio in critically ill patients with cirrhosis: Insights from MIMIC-IV database
Source: PLoS One. 2025 Oct 13;20(10):e0334591. doi: 10.1371/journal.pone.0334591 (PMC12517503; doi:10.1371/journal.pone.0334591)

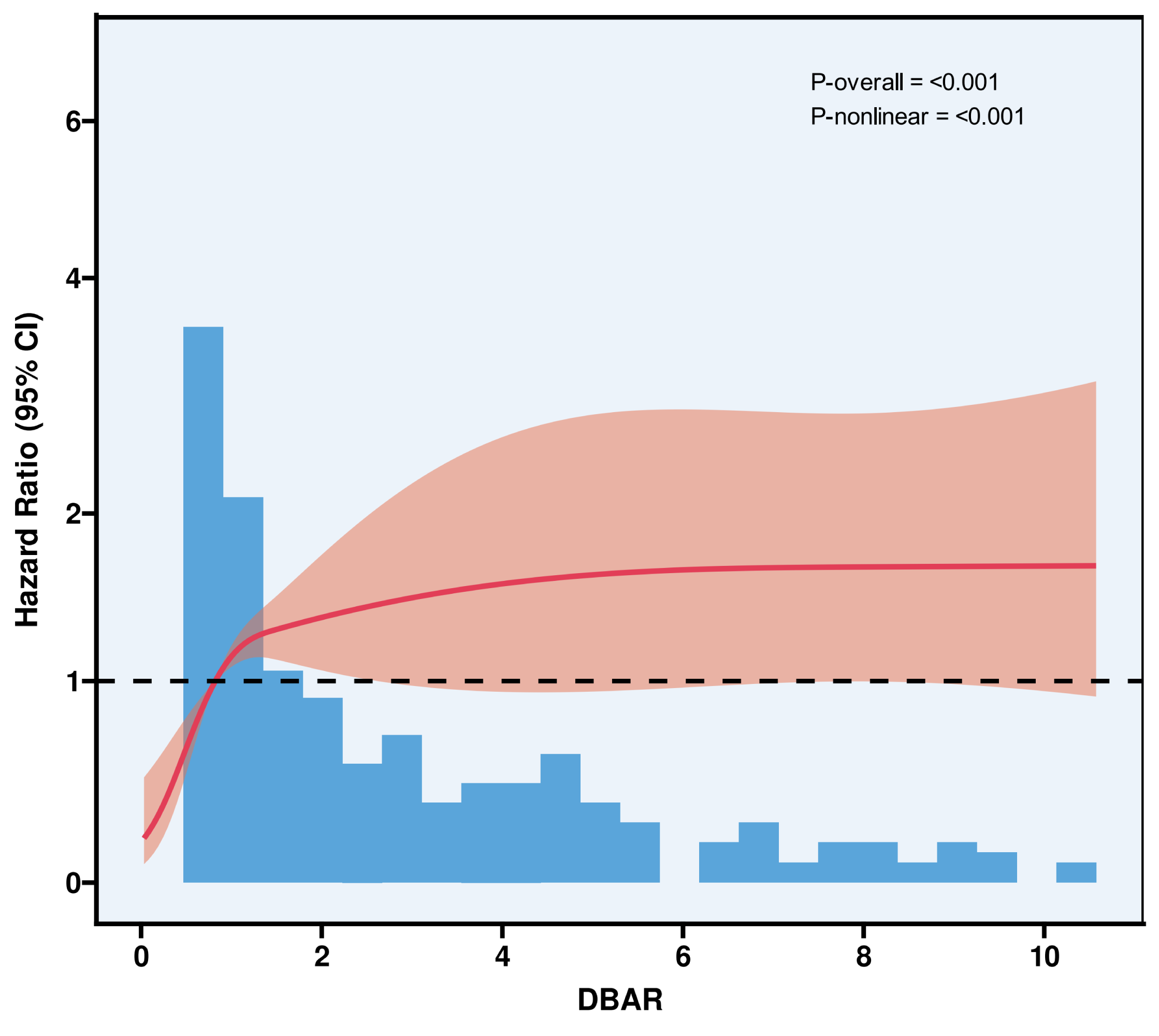

Supplement: S1 Fig — (TIF) [file pone.0334591.s003.tif]

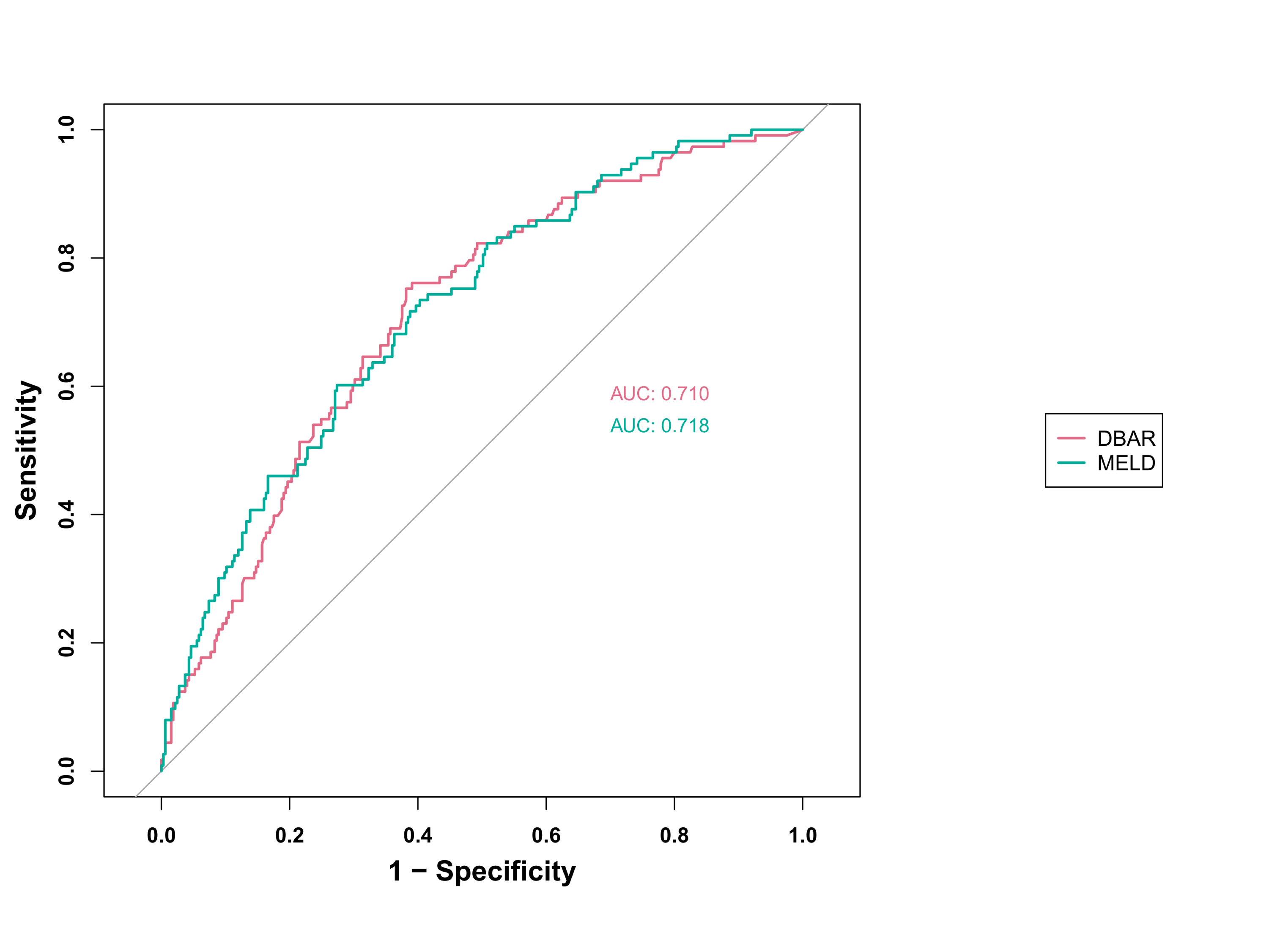

Supplement: S2 Fig — (TIF) [file pone.0334591.s004.tif]
