# Supplementary material for: The prognostic value of the direct bilirubin to albumin ratio in critically ill patients with cirrhosis: Insights from MIMIC-IV database
Source: PLoS One. 2025 Oct 13;20(10):e0334591. doi: 10.1371/journal.pone.0334591 (PMC12517503; doi:10.1371/journal.pone.0334591)
Supplement: Attachment — Submitted filename: Review of Article-20250509.docx [file pone.0334591.s005.docx]

**Summary:**

This study investigates the relationship between the direct bilirubin-to-albumin ratio (DBAR) and 28-day mortality in critically ill patients with cirrhosis. A total of 509 patients were analyzed, with in-hospital and ICU mortality rates of 22.3% and 14.3%, respectively. Both uni-variate and multivariate analyses identified DBAR as an independent risk factor for 28-day mortality (HR = 1.16; 95% CI: 1.10–1.24; p < 0.001). ROC analysis showed DBAR's moderate predictive ability (AUC = 0.702), and patients with DBAR ≥ 4 had significantly higher mortality risk (HR = 3.05; 95% CI: 1.87–4.97). Kaplan-Meier survival curves and restricted cubic spline modeling revealed a nonlinear association between DBAR and prognosis (P-nonlinear = 0.022). Subgroup analyses confirmed consistent effects across clinical subgroups. Overall, DBAR serves as a simple and effective marker for short-term mortality prediction in severe cirrhosis, aiding in clinical risk stratification and management. However, there are some issues need to be addressed by the author.

**Comments:**

1. In Line 114-115:“(Further details are delineated in Fig1)”The parenthetical is too awkward and overly formal. Just rephrase as:“Further details are shown in Figure 1.”
2. The exclusion of “previous ICU admissions” is vague. Were only first ICU stays during hospitalization included? What if the patient had multiple hospitalizations?
3. Missing data over 30% were dropped, but it's unclear whether this applied to variables or subjects.Were entire patients excluded if any variable exceeded 30% missingness, or were individual variables excluded from analysis?
4. In addition, primary endpoint is 28-day all-cause mortality, but it's unclear how death was defined or verified (hospital record, discharge status, etc.).
5. Boruta Feature Selection is introduced but not explained well in context. Please give more explanation for that.
