# Supplementary material for: The prognostic value of the direct bilirubin to albumin ratio in critically ill patients with cirrhosis: Insights from MIMIC-IV database
Source: PLoS One. 2025 Oct 13;20(10):e0334591. doi: 10.1371/journal.pone.0334591 (PMC12517503; doi:10.1371/journal.pone.0334591)
Supplement: Attachment — Submitted filename: Response to Reviewers.docx [file pone.0334591.s006.docx]

Dear Editor and Reviewers,

We sincerely thank you for giving us the opportunity to revise our manuscript titled **“The prognostic value of the direct bilirubin to albumin ratio in critically ill patients with cirrhosis: Insights from MIMIC-IV Database** ”. We greatly appreciate the time and effort both the editor and reviewers have dedicated to reviewing and suggesting improvements to our work. Truly, these invaluable comments and recommendations have assisted us in enhancing the quality of our manuscript. In response, we have made the necessary revisions. We have uploaded two versions of the manuscript: a "Revised Manuscript with Track Changes" and a "Cleaned Revised Manuscript". In the "Revised Manuscript with Track Changes" version, we have highlighted the changes in red for easy identification. Enclosed within this submission, we've provided a point-by-point response to each of the comments raised. The comments from the editor or reviewers are presented in blue italics. We genuinely hope that with these modifications, our manuscript now aligns with the standards of your esteemed journal.

**Reviewer 1**

*This study provides solid evidence that DBAR is an independent predictor of short-term mortality in critically ill cirrhosis patients, though some areas—like variable selection —need clearer explanation.*

**The author's response:**

Thank you for your valuable comments regarding the selection of covariates. To enhance the accuracy and clinical relevance of our model, we included several covariates in Model 3, which are detailed as follows:

**Basic Demographic Information**: We first included basic demographic variables such as age, gender, and race, which are important for assessing the prognosis of critically ill cirrhotic patients.

**Significant Variables from Univariate Cox Regression**: In the univariate Cox regression analysis, we found several variables to be significantly associated with 28-day mortality, including blood urea nitrogen (BUN), potassium (Potassium), sodium (Sodium), creatinine (Creatinine), lactate (Lactate), white blood cell count (WBC), international normalized ratio (INR), acute kidney injury (AKI), systolic blood pressure (SBP), hepatorenal syndrome (HRS), ascites (Ascites), vasopressor use (Vasoactive), and continuous renal replacement therapy (CRRT). These variables were included in the multivariate regression model to ensure the comprehensiveness and accuracy of the model.

**Clinically Relevant Variables**: In addition to the statistically significant variables from univariate analysis, we also included variables that are clinically considered to be closely related to the prognosis of critically ill cirrhosis patients. These include platelet count (Platelet), variceal bleeding (Variceal bleeding), mechanical ventilation use (Ventilator), spontaneous bacterial peritonitis (SBP), SIRS status (SIRS), and sepsis (Sepsis). These variables are crucial in clinical practice for prognostic assessment and contribute to the model's predictive accuracy.

In summary, the selection of covariates for Model 3 not only considered the statistical significance from the univariate Cox regression analysis but also incorporated clinical relevance to ensure that the model accurately reflects the various factors affecting mortality in critically ill cirrhosis patients. We believe that this approach enhances the clinical significance of the model and provides a more clinically useful prognostic tool.

Thank you once again for your meticulous review. We believe these adjustments will further improve the scientific rigor and clinical value of our study.

***Cleaned*** ***Revised Manuscript, Page7, lines 143-146: Page12, lines 211-213***

**2.4 Statistical analyses**

Continuous data were expressed as mean ± SD (normal distribution) or median (IQR) (non-normal distribution), and categorical variables as frequencies (percentages). Baseline characteristics were compared using Student’s t-test, ANOVA, Pearson’s chi-square, or Fisher’s exact test. Data with missing values exceeding 20% were deleted, while data with missing values less than 20% were subjected to random forest imputation. Univariate Cox regression analysis was conducted to identify significant variables, which were then included in the multivariate Cox regression model based on clinical relevance and statistical significance.

**3.3 Relationship between DBAR and 28-day mortality**

In the multivariate Cox regression analysis, variables were selected for inclusion based on their statistical significance in univariate analysis or their established clinical relevance to ensure accurate model adjustment. In the initial model (Model 1), the HR for the association between DBAR and 28-day mortality was 1.21 (95% CI: 1.15–1.26; p<0.001).

*subgroup interpretation—need clearer explanation*

**The author's response:**

Thank you for your thoughtful comments regarding the subgroup analysis. We have already addressed the variability in the predictive efficacy of DBAR among patients with ascites and hepatorenal syndrome (HRS) in the Discussion section. We explained that this variability may be attributed to two primary factors: renal sodium retention and reduced effective circulating blood volume in patients with severe ascites, and the potential interaction effects introduced by aggressive clinical interventions (such as paracentesis and albumin supplementation) in these patients.

We believe these factors are important for explaining the differences in DBAR's predictive accuracy across subgroups. We have further elaborated on these mechanisms and their impact on prognosis in the Discussion section. We appreciate the reviewer’s careful review.

***Cleaned*** ***Revised Manuscript, Page11-12, lines 241-255:***

In this study, univariate and multivariate prognostic analyses were performed on a cohort of critically ill cirrhotic patients, revealing that the DBAR serves as an independent predictor of short-term all-cause mortality. Patients were categorized into high-risk (DBAR ≥4) and low-risk (DBAR <4) groups based on DBAR thresholds. KM survival analysis demonstrated a persistently significant prognostic disparity in the high-risk group, even after adjusting for multiple covariates (HR: 3.05, P<0.001). Both DBAR and the MELD score exhibited moderate discriminative capacity in predicting 28-day mortality, with consistent predictive accuracy for in-hospital (ROC: 0.720) and 90-day mortality (ROC: 0.676). These findings underscore the potential clinical significance of DBAR in evaluating both short- and medium-to-long-term prognoses in critically ill cirrhotic patients. Restricted cubic spline analysis disclosed a statistically significant nonlinear association between DBAR and cirrhosis, with hazard ratios progressively escalating as DBAR values increased. Subgroup analysis revealed variability in the predictive efficacy of DBAR among patients with ascites and HRS. This variability can be attributed to two primary factors: First, severe ascites is frequently associated with renal sodium retention and diminished effective circulating blood volume, which may exacerbate renal dysfunction, disrupt albumin metabolism, and impair bilirubin clearance. Second, the imperative for aggressive interventions, such as paracentesis and albumin supplementation, in these patients may introduce interaction effects that influence prognosis. This study elucidates the potential role of DBAR as a prognostic biomarker in critically ill cirrhotic patients, while highlighting the complex interplay of pathophysiological mechanisms in advanced liver disease.

*Also, the sentences in the context could be improved for clarity, flow, and scientific rigor.*

**The author's response:**

Thank you for your thoughtful review and valuable suggestions. We have revised the sentences in the manuscript to improve clarity, flow, and scientific rigor, as per your recommendation. The revised text has also been carefully polished by a native English speaker to ensure language accuracy. We believe these changes enhance the readability and accuracy of our findings. We greatly appreciate your constructive feedback.

**Reviewer 2**

1. *The authors should state clearly in abstract that the patient data is from public repository*

**The author's response:**

We sincerely thank the reviewer for this valuable suggestion. In accordance with the comment, we have revised the Background section of the abstract to explicitly state that the patient data were obtained from the publicly available MIMIC-IV database.

***Cleaned*** ***Revised Manuscript, Page11-12, lines 241-255:***

Patients with severe cirrhosis are at a higher risk of mortality. This study aimed to investigate the association between the direct bilirubin-to-albumin ratio (DBAR) and 28-day mortality in critically ill cirrhosis patients using data from the publicly available MIMIC-IV database.

*2. Upon univariate or multivariate analysis, there were other variables that are also significantly different between the two groups, why did the authors finally focus on DBAR but not others? Have the authors compared other variables like lactate or BUN with DBAR in terms of AUC or cubic spline analysis? Clearly from Fig.4, Lactate or BUN had the higher importance score than DBAR.*

**The author's response:**

We sincerely thank the reviewer for this valuable comment. Indeed, biomarkers such as lactate and BUN have been widely studied as prognostic indicators in cirrhotic patients. For example, Krispin et al. (2023) reported that an elevated lactate-to-albumin ratio is a novel predictor of in-hospital mortality in hospitalized cirrhotics (Ann Hepatol. 2023;28(3):100897. PMID: 36632976). Similarly, Shen and Shan (2023) demonstrated that the blood urea nitrogen-to-albumin ratio (BAR) is an independent predictor of poor outcomes in HBV-related decompensated cirrhosis (Clin Lab. 2023;69(4). PMID: 37057953).

These studies underscore the established clinical relevance of lactate and BUN (particularly BAR). In contrast, the direct bilirubin-to-albumin ratio (DBAR) has not been systematically evaluated in this population. Therefore, our study focused on DBAR to fill this gap and to provide complementary evidence to the existing literature.

Although Fig. 4 indicates that lactate and BUN had higher importance scores, our analyses demonstrated that DBAR remains an independent predictor of short-term mortality, with reasonable discriminative ability. Importantly, DBAR combines two liver-related indicators (bilirubin and albumin), reflecting both hepatic function and nutritional/inflammatory status, which makes it a convenient and biologically meaningful tool for clinical practice.

Thus, our intention was not to replace lactate or BUN, but rather to propose DBAR as a novel and practical prognostic marker that can supplement traditional predictors in critically ill cirrhosis patients.

*3. The authors may consider validating the important variables in other cohorts to corroborate the finding.*

**The author's response:**

We sincerely thank the reviewer for this important suggestion. In order to further validate the robustness of our findings, we performed an external validation using the eICU Collaborative Research Database (eICU-CRD). The eICU-CRD is a large, publicly available, multicenter critical care database that contains de-identified health records of over 200,000 ICU admissions from diverse hospitals across the United States. Consistent with the results from the MIMIC-IV cohort, DBAR remained an independent predictor of short-term mortality in the eICU-CRD population after adjustment in multivariable Cox regression models. In addition, restricted cubic spline (RCS) analysis confirmed a nonlinear dose–response association, and ROC curve analysis demonstrated good discriminative ability (AUC ≈ 0.71). Taken together, these findings further corroborate the robustness and generalizability of DBAR as a prognostic marker in critically ill cirrhosis patients.

Association between DBAR and survival (Cox regression analysis based on the eICU-CRD database)

| **Characteristic** | **Model 1** | | | **Model 2** | | | **Model 3** | | |
| --- | --- | --- | --- | --- | --- | --- | --- | --- | --- |
|  | **HR** | **95% CI** | **p-value** | **HR** | **95% CI** | **p-value** | **HR** | **95% CI** | **p-value** |
| **DBAR (continuous)** | 1.13 | 1.08, 1.19 | <0.001 | 1.12 | 1.06, 1.18 | <0.001 | 1.07 | 1.00, 1.15 | 0.038 |
| **DBAR** |  |  |  |  |  |  |  |  |  |
| Q1 [0,0.305) | — | — |  | — | — |  | — | — |  |
| Q2 [0.305,0.821) | 2.39 | 1.07, 5.35 | 0.034 | 2.48 | 1.10, 5.58 | 0.028 | 3.02 | 1.29, 7.12 | 0.011 |
| Q3 [0.821,2.69) | 5.36 | 2.57, 11.18 | <0.001 | 5.25 | 2.51, 10.98 | <0.001 | 4.36 | 2.00, 9.50 | <0.001 |
| Q4 [2.69,17.6] | 7.89 | 3.77, 16.52 | <0.001 | 7.36 | 3.51, 15.46 | <0.001 | 5.12 | 2.21, 11.87 | <0.001 |
| P for trend |  |  | <0.001 |  |  | <0.001 |  |  | <0.001 |
| Abbreviations: CI = Confidence Interval, HR = Hazard Ratio | | | | | | | | | |
| Model 1 : adjusted for Age, Gender, and Race  Model 2 : adjusted for Age, Gender, Race, BUN, and Cr  Model 3 : adjusted for Age, Gender, Race, Platelet, WBC, Potassium, Sodium, Lactate, INR, BUN, Cr, SBP, Ascite, Varicealbleeding, HRS, Sepsis, AKI, MV, CRRT, and vasopressor | | | | | | | | | |

**Figure-ROC (eICU-CRD database)**

**Figure-RCS (eICU-CRD database)**
