# Supplementary material for: The prognostic value of the direct bilirubin to albumin ratio in critically ill patients with cirrhosis: Insights from MIMIC-IV database
Source: PLoS One. 2025 Oct 13;20(10):e0334591. doi: 10.1371/journal.pone.0334591 (PMC12517503; doi:10.1371/journal.pone.0334591)
Supplement: Attachment — Submitted filename: Response_to_Reviewers_auresp_2.docx [file pone.0334591.s007.docx]

Dear Editor and Reviewers,

We sincerely thank you for giving us the opportunity to revise our manuscript titled **“The prognostic value of the direct bilirubin to albumin ratio in critically ill patients with cirrhosis: Insights from MIMIC-IV Database** ”. We greatly appreciate the time and effort both the editor and reviewers have dedicated to reviewing and suggesting improvements to our work. Truly, these invaluable comments and recommendations have assisted us in enhancing the quality of our manuscript. In response, we have made the necessary revisions. We have uploaded two versions of the manuscript: a "Revised Manuscript with Track Changes" and a "Cleaned Revised Manuscript". In the "Revised Manuscript with Track Changes" version, we have highlighted the changes in red for easy identification. Enclosed within this submission, we've provided a point-by-point response to each of the comments raised. The comments from the editor or reviewers are presented in blue italics. We genuinely hope that with these modifications, our manuscript now aligns with the standards of your esteemed journal.

**Reviewer**

*Given the consistency between the results obtained from MIMIC-IV database and the validation cohort eICU-CRD, the authors should incorporate the results shown in replies to reviewers' comments into the manuscript to be a separate figure.*

**The author's response:**

We sincerely thank the reviewer for this valuable suggestion, which has greatly improved the quality of our manuscript and provided us with significant insights. In accordance with your advice, we have incorporated the external validation results from the eICU-CRD cohort into the main text and presented them in a separate section with supplementary figures.

***Cleaned*** ***Revised Manuscript, Page17-19, lines 287-317; Page20, lines 348-351***

**3.8 External validation using eICU-CRD cohort**

To validate the robustness of our findings, we further analyzed an independent validation cohort from the eICU Collaborative Research Database (eICU-CRD), a large multicenter critical care database comprising over 200,000 ICU admissions across the United States. Consistent with the results from the MIMIC-IV cohort, DBAR remained an independent predictor of short-term mortality in the eICU-CRD population after adjustment in multivariable Cox regression models. RCS analysis confirmed a nonlinear dose–response association between DBAR and short-term mortality risk. Moreover, ROC curve analysis demonstrated good discriminative ability, with an AUC of approximately 0.71 . These findings further corroborate the robustness and generalizability of DBAR as a prognostic biomarker in critically ill cirrhotic patients.

Importantly, the prognostic value of DBAR was further confirmed in an independent external validation cohort (eICU-CRD), underscoring its robustness and generalizability across diverse ICU populations.

The restricted cubic spline analysis and ROC analysis of the validation cohort are provided as **Supplementary Fig 1** and **Fig 2**. The detailed results of the multivariable Cox regression models are presented in Supplementary **Table 1**.

| **Variables** | **Model 1** | | | **Model 2** | | | **Model 3** | | |
| --- | --- | --- | --- | --- | --- | --- | --- | --- | --- |
|  | **HR** | **95% CI** | **p-value** | **HR** | **95% CI** | **p-value** | **HR** | **95% CI** | **p-value** |
| DBAR (continuous) | 1.13 | 1.08, 1.19 | <0.001 | 1.12 | 1.06, 1.18 | <0.001 | 1.07 | 1.00, 1.15 | 0.038 |
| DBAR |  |  |  |  |  |  |  |  |  |
| Q1 [0,0.305) | 1(Reference) | | | 1(Reference) | | | 1(Reference) | | |
| Q2 [0.305,0.821) | 2.39 | 1.07, 5.35 | 0.034 | 2.48 | 1.10, 5.58 | 0.028 | 3.02 | 1.29, 7.12 | 0.011 |
| Q3 [0.821,2.69) | 5.36 | 2.57, 11.18 | <0.001 | 5.25 | 2.51, 10.98 | <0.001 | 4.36 | 2.00, 9.50 | <0.001 |
| Q4 [2.69,17.6] | 7.89 | 3.77, 16.52 | <0.001 | 7.36 | 3.51, 15.46 | <0.001 | 5.12 | 2.21, 11.87 | <0.001 |
| P for trend |  |  | <0.001 |  |  | <0.001 |  |  | <0.001 |

**Supplementary Table 1:** Association between DBAR and mortality in the eICU-CRD validation cohort.

Model 1 : adjusted for age, gender, and race ;

Model 2 : adjusted for age, gender, race, bun, creatinine ;

Model 3 : adjusted for age, gender, race, bun, creatinine, potassium, sodium, lactate, WBC, platelet, INR, Sepsis, AKI, Spontaneous bacterial peritonitis, Hepatorenal syndrome, Variceal bleeding, Ascites, Vasoactive, Ventilator, and CRRT

**Supplementary Fig 1** **:** DBAR and mortality in the eICU-CRD cohort (RCS analysis).

**Supplementary Fig 2 :** DBAR and mortality in the eICU-CRD cohort (ROC analysis).

**4 Discussion**

Importantly, the prognostic significance of DBAR was further confirmed in an independent external validation cohort (eICU-CRD), which underscores the robustness and generalizability of our findings across diverse ICU populations. This study elucidates the potential role of DBAR as a prognostic biomarker in critically ill cirrhotic patients, while highlighting the complex interplay of pathophysiological mechanisms in advanced liver disease.
